# Malacards: The Human Disease Database

**DOI:** 10.5195/jmla.2018.253

**Published:** 2018-01-02

**Authors:** Sue Espe

MalaCards: The Human Disease Database is a robust database designed to enable genomic and genetic researchers, investigators, and scholars to efficiently navigate the universe of human genes, genetic variants, proteins, cells, and biological pathways related to various human diseases. Available since 2013, MalaCards provides detailed data on thousands of human diseases and their relationships with respective genes. Rappaport et al. describe the MalaCards project as an “attempt to generate a complete lexicon of all human diseases” [1]. MalaCards is part of the LifeMap Sciences suite of products that includes the GeneCards Suite product line and contains relevant, integrated content from its genetics product line, such as GeneCards and GeneAnalytics.

Each disease is arranged in MalaCards under 2 distinct categories: anatomical and global. The anatomical disease section includes 18 major categories with more than 26,000 diseases representing all areas of the body, including blood, bone, immune, muscle, and reproductive diseases. The global disease section contains 6 categories: cancer, fetal, genetic, infectious, metabolic, and rare diseases, and almost 18,000 specific diseases.

The disease names have been compiled from 10 primary disease databases with another 11 databases as secondary sources. Overall, the site includes over 19,000 disease entries, which correspond with more than 13,000 genes from approximately 70 prominent databases and websites. Unique entries number close to 13,000 [1]. MalaCards uses several medical classification schemes, language systems, and related ontologies. Genetic information, variations, and associated information related to the disease are included along with annotations, matrices, and mapped tables.

## NAVIGATION

The main navigation through the MalaCards website begins on the home page, which includes six tabs: User Guide, Analysis Tools, News and Views, Disease Lists/Categories, and About. You can find diseases by selecting a category from the Disease Lists/Categories tab and browsing the resulting list of diseases. The interrelated characteristics of diseases are illustrated on each disease page. In addition, the GeneCards Suite of databases can be accessed from the MalaCards home page.

## SEARCH

MalaCards allows both simple keyword searches and advanced searches. With the advanced search, you can search eleven pre-defined items, including Name, Aliases, Expressions, and Variations. You can use wildcard characters for truncation, search using the Boolean operators “AND” and “OR,” build nested Boolean searches, and search phrases by enclosing the phrase in quotation marks. Results for exact matches as well as related terms are displayed in the results. An alpha-numeric index is also provided to search by specific disease name.

A MalaCards search retrieves a “disease card” with substantial detailed data about the searched disease. On this page, the header notes all the categories in which the disease topic lies, and the remainder of the page contains the data from each of the fifteen sections. Examples of specific data found in the sections include top genes, mutations, significance, phenotypes, clinical trials, [Chemical Abstracts Service] CAS registry number, and PubChem Id. Each disease card contains fourteen sections: Aliases & Classifications, Anatomical Context, Drugs & Therapeutics, Expression, Genes, Genetic Tests, [Gene Ontology] GO Terms, Pathways, Publications, Related Diseases, Sources, Summaries, Symptoms and Phenotypes, and Variations.

Before using MalaCards, you should read through the material under the User Guide tab to learn more about MalaCards’ organization, schemes, and classifications. Although the search features are fairly standard, some features may be hard to interpret. For instance, headings in MalaCards can contain a unique string of text and numbers that has a particular association for identification purposes. These strings—along with other features unique to MalaCards—are explained in the User Guide.

Additional information about MalaCards can be found in the three published manuscripts found in the references section of this review. These articles include in-depth information about its disease-naming methods, data mining techniques, and content. MalaCards data are obtained through legitimate, authoritative sources, but users must be aware that MalaCards is “automatically generated from publicly available data, and therefore is only as accurate as the information on which it is based. Different disease granularity, as well as diverse conventions and medical definitions, may cause redundancy and reduce integration” [2].

MalaCards developers routinely monitor data authenticity, accuracy, and integrity and perform fixes or enhancements at least three times a year. Overall, the developers maintain that “quality assurance is instituted on every MalaCards update and version” [3]. Unified and utilizing multiple database sources, MalaCards provides researchers and clinicians with a powerful resource to search numerous diseases, particularly as they relate to genetics and genomics.
